# Permanent control of HIV-1 pathogenesis in exceptional elite controllers: a model of spontaneous cure

**DOI:** 10.1038/s41598-020-58696-y

**Published:** 2020-02-05

**Authors:** Concepcion Casado, Cristina Galvez, Maria Pernas, Laura Tarancon-Diez, Carmen Rodriguez, Víctor Sanchez-Merino, Mar Vera, Isabel Olivares, Rebeca De Pablo-Bernal, Alberto Merino-Mansilla, Jorge Del Romero, Ramon Lorenzo-Redondo, Ezequiel Ruiz-Mateos, María Salgado, Javier Martinez-Picado, Cecilio Lopez-Galindez

**Affiliations:** 1 Virología Molecular, Laboratorio de Referencia e Investigación en Retrovirus, Centro Nacional de Microbiología, Instituto de Salud Carlos III, Majadahonda, Madrid Spain; 2 AIDS Research Institute IrsiCaixa, Badalona, Spain; 3 Universitat Autònoma de Barcelona, Cerdanyola del Vallès, Spain; 4 Clinical Unit of Infectious Diseases, Microbiology and Preventive Medicine, Institute of Biomedicine of Seville (IBiS), Virgen del Rocío University Hospital, CSIC, University of Seville, Seville, Spain; 5 Centro Sanitario Sandoval, Hospital Clínico San Carlos. IdISSC, Madrid, Spain; 6 AIDS Immunopathology Unit. Laboratorio de Referencia e Investigación en Retrovirus. Centro Nacional de Microbiología, Instituto de Salud Carlos III, Majadahonda, Madrid Spain; 7 Division of Infectious Diseases, Northwestern University Feinberg School of Medicine, Chicago, Illinois 60011 USA; 8 University of Vic-Central University of Catalonia (UVic-UCC), Vic, Spain; 9 Catalan Institution for Research and Advanced Studies (ICREA), Barcelona, Spain

**Keywords:** Evolution, Biomarkers, Diseases, Molecular medicine, Pathogenesis

## Abstract

Elite controllers (EC) represent a small subset of HIV-1-infected people that spontaneously control viral replication. However, natural virological suppression and absence of immune dysfunction are not always long-term sustained. We define exceptional EC (EEC) as HIV-1 subjects who maintain the EC characteristics without disease progression for more than 25 years. We analyzed three EEC, diagnosed between 1988 and 1992, who never showed signs of clinical disease progression in absence of any antiretroviral treatment. A comprehensive clinical, virological, and immunological study was performed. The individuals simultaneously exhibited ≥3 described host protective alleles, low levels of total HIV-1 DNA (<20 copies/10^6^ CD4^+^ T-cells) without evidence of replication-competent viruses (<0.025 IUPM), consistent with high levels of defective genomes, strong cellular HIV-1-specific immune response, and a high poly-functionality index (>0.50). Inflammation levels of EEC were similar to HIV-1 negative donors. Remarkably, they showed an exceptional lack of viral evolution and 8-fold lower genetic diversity (<0.01 s/n) in *env* gene than other EC. We postulate that these EEC represent cases of spontaneous functional HIV-1 cure. A non-functional and non-genetically evolving viral reservoir along with an HIV-1-specific immune response seems to be key for the spontaneous functional cure.

## Introduction

The use of combination antiretroviral therapy (ART) results in sustained undetectable plasma viremia in HIV-1-infected individuals. The success of ART led to initial optimism that HIV-1 may be cured by ART alone; however subsequent studies indicating that such a cure may take as long as 70 years^1,2^ led researchers to attempt complementary strategies to reduce viral persistence and potentially facilitate HIV-1 remission^3^. However, this has proven extremely difficult to achieve because it entails the eradication of any infectious viral form from the body. This has only putatively been achieved so far in two individuals, the Berlin and London patients^4,5^. A less stringent objective, known as functional cure, consists in the permanent suppression of HIV-1 viral replication in the absence of ART even if full viral eradication is not achieved^6^.

Elite controllers (EC) represent a small proportion of HIV-infected patients with a spontaneous control of HIV replication at undetectable levels. However, they are heterogeneous in terms of long-term clinical, virological and immunological progression. Among them, there is a subgroup of subjects who have an asymptomatic HIV-1 infection and prolonged control of clinical progression without ART. The closest cases of spontaneous functional HIV-1 cure are potentially represented by this clinical phenotype of slow or no disease progression^7^. The underlying mechanisms contributing to this control include host and viral factors^8,9^. Our previous work stablished differences in EC with long-term non-progression compared to EC that ultimately lost control^10^. Previous reports have described several selected cases of durable control over HIV-1 replication without ART^11,12^; however, the cases described herein are Exceptional Elite Controllers (EEC) with no disease progression for more than 25 years. The question whether these individuals can be considered cured remains unresolved. Also, there is a need for differential markers that can characterize these unique cases of potential HIV-1 remission.

In this study, we characterized three EEC diagnosed for 25, 28 and 29 years with an extensive follow-up, permanent control of viral replication, and no sign of disease progression. We investigated these individuals to determine if they can be considered spontaneous cases of HIV-1 functional cure. Multiple host genetic, immunological, and virological factors were examined to distinguish this extraordinary phenotype and to identify potential diagnostic markers.

## Materials and Methods

### Individuals, samples and clinical characteristics

The study individuals were selected from a previous long-term EC cohort^9^. These subjects have been followed in the Centro Sanitario Sandoval (a primary sexual clinic without antiretroviral prescription belonging to the Hospital Clínico San Carlos. IdISSC, Madrid) for more than 20 years. All individuals were diagnosed with a second-generation ELISA (Abbot) and confirmed by Western-Blot (BioRad) after 5 to 6 years of the estimated infection. Participants gave informed consent for molecular and genetic studies. In March 2017, 500 mL of peripheral blood was drawn to perform a comprehensive analysis of multiple immunological and virological markers. All clinical investigations were conducted according to the principles expressed in the Declaration of Helsinki. The study was approved by the Research Ethics and Animal Welfare Committee of the Instituto de Salud Carlos III (CEI PI 05_2010-v3) and by the Ethics and Clinical Research Committee of the Hospital Clínico San Carlos in Madrid (Number C.I. 16/490-E).

### Viral persistence

#### Total proviral HIV-1 DNA

Total proviral reservoir was quantified in two samples, the 2017 and another 11 to 13 years before. Purified peripheral CD4^+^ T cell-lysed (Miltenyi Biotec, Germany) extracts were used for total HIV-1 DNA by droplet-digital polymerase chain reaction (ddPCR)^13^. To circumvent sequence mismatches in viral sequences, two primer sets in the viral 5′ long terminal repeat (5-LTR) and *gag* regions were employed. The RPP30 housekeeping gene was quantified to normalize sample input. We analyzed 1.5–1.8 million cells in several replicates, with a limit of detection of (LOD) 1–3 copies/10^6^ CD4^+^ T cells. Raw ddPCR data were examined using the QX100™.

#### Quantitative Viral Outgrowth Assay (qVOA)

The replication-competent reservoir was measured on fresh CD4^+^ T cells from each HIV-1 individual in 28–63 × 10^6^ CD4^+^ T cells in a limiting dilution cell culture assay^13^. Supernatants from day 14 were quantified with p24^Gag^ ELISA (Perkin-Elmer, USA). Infectious HIV-1 units per million (IUPM) CD4^+^ T cells were determined using IUPMStats v.1 (https://silicianolab.johnshopkins.edu/) based on the maximum likelihood method.

#### Plasma viral load and residual viremia

Viral load was determined with the Quantiplex HIV-1 RNA 2.0, 3.0 and the Versant NA (Siemens, Germany) with different limits of detection. For residual viremia determination, nine mL of plasma were ultracentrifuged at 170,000 g at 4 °C for 30 minutes before HIV-1 RNA quantification using the Abbott Real-Time HIV-1 assay (Abbott, Ill, USA)^4,14^ with a LOD of 0.5 copies/mL.

#### Cell-Associated HIV-1 RNA

Viral transcription was evaluated by quantification of cell-associated HIV-1 RNA in purified CD4^+^ T cells by a one-step reverse-transcription ddPCR (Bio-Rad, USA) with primers and probe in the viral 5-LTR and *gag* gene, and in the housekeeping gene of TATA-binding protein (TBP)^13^. Samples were analyzed on several replicates with 3–5 million cells for each subject. Raw ddPCR data were analyzed using the QX100™ Droplet Reader and the QuantaSoft v.1.6 software (Bio-Rad, USA) with a variable LOD between 0.03 and 0.05 (ratio HIV/TBP).

### Genetic studies

#### Nucleotide sequence and phylogenetic analysis in *env* gene

Sequences were obtained by limiting dilution in a 614 bp C2–V5 fragment in *env* gene^15^. All sequences with gaps and hypermutation (only two in one sample from EEC-3) were excluded of analysis. Near full-length genome analysis was performed with overlapping limiting dilution PCR^16^. Viral dating of the individual sequences was carried out according to^17^. Co-receptor usage was predicted with WebPSSM tool (https://indra.mullins.microbiol.washington.edu/webpssm/) using the SINSI scores.

All nucleotide sequences generated in this study were submitted to GenBank under accession numbers: AY501160–AY501179, AY501254–AY501269, KC595083–KC595089, KC595099–KC595105, KC595118–KC595119, EU644051, EU644056–EU644060, MH595843, MH595844, MH595846, MN068093–MN068211 and MN055643.

#### Quasispecies diversity analysis

We selected sequences from individual quasispecies in the HIV-1 database (http://hiv.lanl.gov/) from the same region analyzed in the study. We included infected individuals with undetectable viral load under prolonged ART (range [2–15 years])^18,19^, long-term viremic non progressors (LTNPs) with more than 10 years of infection and transient EC^10^ defined as individuals that lost spontaneous viral control during the follow-up, for comparison with the study subjects. We estimated the average diversity over sequence pairs for each time point in every individual and calculated the average number of base differences per site (p-distance) with 500 bootstrap replicates using MEGA6. All positions containing gaps and missing data were eliminated. Differences in the average p-distance per individual between groups were tested for significance using pairwise Wilcoxon Rank-Sum Test in R v3.5.0, applying the Benjamini-Hochberg procedure for multiple comparisons correction (cut off for significance False Discovery Rate 0.05).

### Immune responses

#### Cell stimulation

Peripheral blood mononuclear cells (PBMCs) were thawed, washed and *in vitro* stimulated with overlapped HIV-1 (Gag)-specific peptide pool (NIH AIDS Research and Referenced Reagent Program) as described^10,20^.

#### Immunophenotyping and intracellular cytokine staining

Stimulated PBMCs were stained with surface and intracellular marker antibodies (Supplementary Information Fig. 1)^21^. For dendritic cell immunostaining, myeloid dendritic cells (mDCs) and plasmacytoid dendritic cells (pDCs) were defined as Lin2-HLA-DR + CD11c + CD123- and Lin2-HLA-DR + CD11c-CD123 + , respectively^21^. PBMCs were analyzed in a LSR Fortessa Cell Analyzer (BD Biosciences, Spain).

### Susceptibility and viral inhibition assay

CD4^+^ T cells alone or in combination with autologous CD8^+^ T cells from each patient were infected by spinoculation with 40 ng of p24^Gag^ of the laboratory viral strain HIV-1_NFN-SX_ (CCR5 tropic), and HIV-1_NL4–3_ (CXCR4 tropic). CD4^+^ T cells were activated for 3 days with PHA and IL-2 before infection. Supernatants were sampled at day 7 and quantified with HIV-1 p24^Gag^ ELISA (Perkin-Elmer life Sciences, USA).

### Assay of soluble biomarkers

Plasma samples were collected in EDTA-lined tubes, and aliquoted and stored at −20 °C. The levels of high sensitivity C-reactive protein (hsCRP) and β2-microglobulin (β2M), D-dimer, IL-6 and sCD163 were performed as described^22^.

### HIV-1 antibody determination

Plasma antibody responses were measured in two samples, the 2017 one and another 14 to 19 years before for comparison with two long term viremic controllers as controls (#12 and #60) with a follow-up of >10 years with > 500 cells/µl and VL >2,000 cp/µl. End-point plasma titers of HIV-1 antibodies were measured in an enzyme immunoassay (Genscreen™ HIV-1/2 Version 2, Bio-Rad). Antibodies against the HIV-1 proteins were determined by Western Blot (Biokit, Werfen, Spain) and Line Immuno Assay (InnoLia, Fujirebio Europe N.V, Belgium) and quantified with the LiRAS software. Neutralizing HIV-1 antibody titers in purified IgGs were tested against a mini panel of isolates^23^.

### Statistical analysis

Changes over time in total HIV-1 DNA were evaluated using the Wilcoxon signed rank test. Gag-specific T-cell response poly-functionality was quantified with the poly-functionality index algorithm^24^ employing 0.1.2 beta version of the “FunkyCells Boolean Dataminer” software (www.FunkyCells.com) provided by Dr. Martin Larson (INSERM U1135, France). Analyses were performed with Prism 7 (GraphPad, USA) and the statistical significance was set at 5% for all tests.

## Results

### Clinical characteristics of the individuals

**Figure 1:**
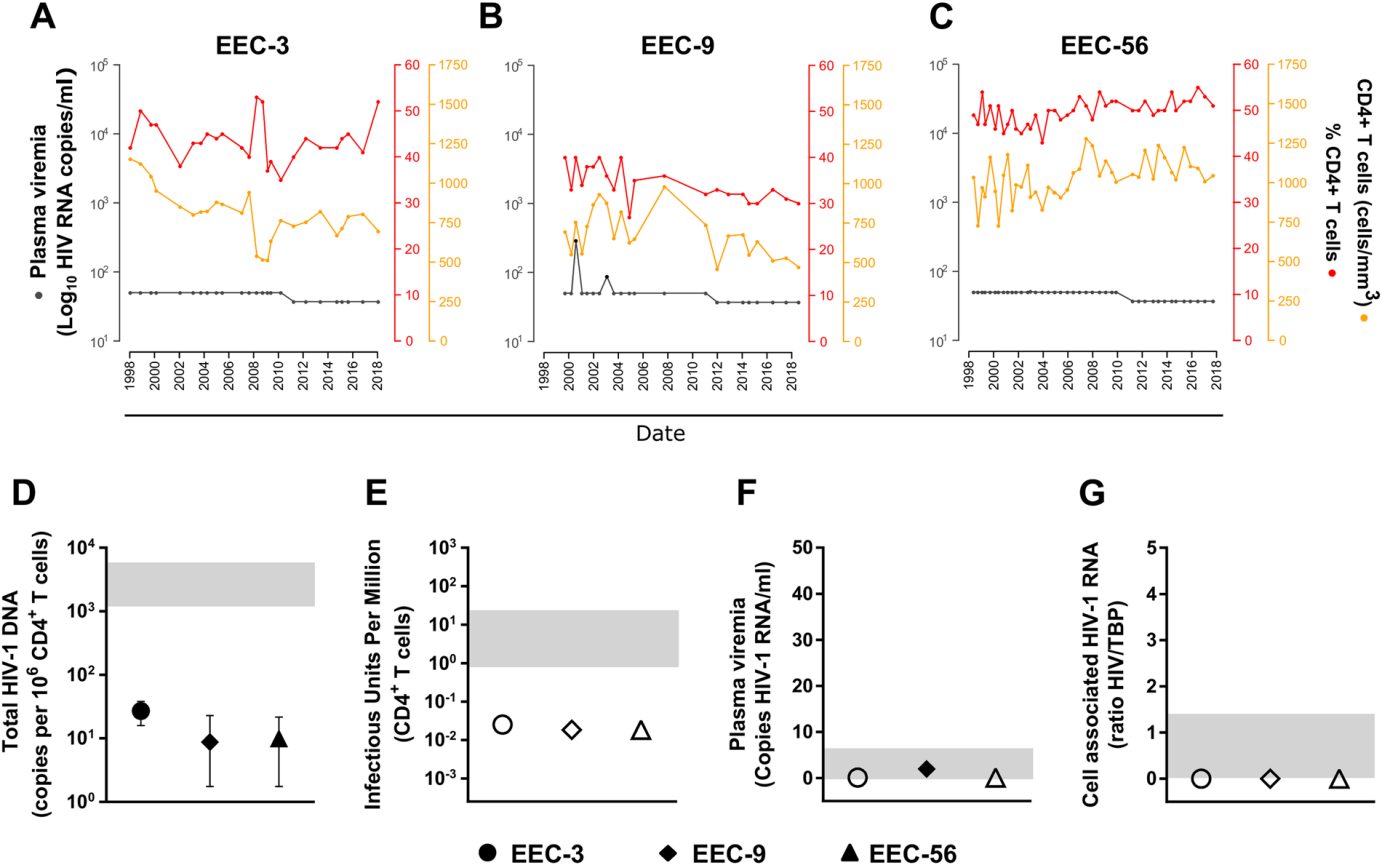
Clinical characteristics and HIV-1 reservoir quantification. (**A–C**) Plasma RNA viral load, absolute CD4^+^ and CD4^+^ T cell percentage over time in the individuals studied. Grey symbols for viral load indicate values below the detection limit. (**D**–**G**) Viral reservoir measurements of (**D**) total HIV-1 DNA, (**E**) Infectious Units per million cells (IUPM) in a qVOA assay, (**F**) ultrasensitive plasma viral load and (**G**) cell associated RNA (caHIV-1-RNA). Open symbols indicate undetectable values. Light grey bands are the interquartile range from standard HIV-1-infected individuals under treatment^26^.

The three individuals of the study (EEC-3, EEC-9, and EEC-56) maintained undetectable viral loads (except two early blips in EEC-9) and stable CD4^+^ and CD8^+^ T-cell counts in absence of ART for more than 25 years of infection (Table 1 and Fig. 1A–C). Hepatitis C Virus (HCV) infection was diagnosed in the three individuals because of intravenous drug use (IVDU) and all reported viral clearance, either spontaneously or after treatment (Table 1). The individuals reported no further exposure to HIV-1 since their diagnosis. The two females of the study gave birth uninfected children in absence of any ART. All individuals were classified as clinical status A, with no symptoms of HIV-1 clinical progression or AIDS defining events.

**Table 1:** Clinical, epidemiological and host-genetic characteristics.

	EEC-3	EEC-9	EEC-56
Year of birth	1956	1957	1957
Sex	Male	Female	Female
Race	Caucasian	Caucasian	Caucasian
Estimated year of infectionª	1983 ± 2	1986 ± 2	1984 ± 2
Year of HIV-1 diagnosis	1988	1992	1989
Age at diagnosis	32	35	32
Transmission route^b^	IVDU	IVDU	IVDU
Remarks	—	HIV-1 negative child	HIV-1 negative child
HCV coinfection	1996	1992	1993
Treatment	RBV/IFNα (2008)	Spontaneous clearance	DAA (2017)
Genetic markers			
CCR5 ∆32 rs333^c^	11	11	11
CCR2 V64I rs1799864^c^	11	11	11
HLA C rs9264942 ^c^	22	22	22
HLA A	02:01, 02:05	02:01, 31:01	01:01, 02:01
HLA B	27:05, 58:01	39:01, 57:01	14:02, 57:01
Genetic Score	4	3	4

^a^Viral dating performed in the last sample and estimated according to^17^.

^b^No further expositions after HIV-1 diagnosis.

^c^“1” indicates the most frequent allele and “2” the mutant.

IVDU, intravenous drug user; RBV, ribavirin; IFNα, interferon α, DAA, direct acting antivirals.

Participants showed several host protective haplotypes, including HLA-B*57:01, HLA-B*58:01, HLA-B*27:05 or HLA-B*14:02^9,25^. In addition, all individuals presented the T > C mutation in the HLA-C polymorphism (rs9264942) in homozygosis (Table 1) associated with lower plasma viremia and increased levels of HLA-C expression^25^. All subjects displayed three to four host protective haplotypes and a high genetic score (Table 1)^9,25^.

### Viral persistence

To evaluate the extent of viral persistence in the study subjects we used the most sensitive assays available, and compared with values obtained from samples from individuals on ART^26^. The quantification of HIV-1 DNA in infected CD4^+^ T cells, which are believed to constitute most of the latent reservoir in peripheral blood, showed detectable, albeit extremely low (median of 10.4 [9.1–23.0] copies/10^6^ CD4^+^ T cells) total HIV-1 DNA in all subjects (Fig. 1D). Similar numbers of provirus-containing cells were detected in frozen samples obtained 11 to 13 years earlier (median of 12.5 [IQR 2.5–31.3] copies/10^6^ CD4^+^ T cells) (data not shown) suggesting the stability of the viral reservoir for long periods of time.

As total HIV-1 DNA indistinctly measures both defective and replication-competent forms, we quantified the frequency of IUPM in CD4^+^ T cells in a co-cultured quantitative viral outgrowth assay (qVOA). We did not detect any replication competent virus in a large amount (28–63 million cells) of highly activated CD4^+^ T cells from the individuals, indicating values of IUPM below 0.025 (Fig. 1E).

The ultrasensitive plasma viremia assay only detected 2 copies of HIV-1 RNA per mL of plasma in EEC-9 and in the other two subjects it was undetectable in 9 ml of plasma (Fig. 1F). Quantification of cell-associated HIV-1 RNA (ca-HIV-1 RNA) showed absence of viral transcription in peripheral CD4^+^ T cells in the three subjects in more than 1 million tested cells (Fig. 1G).

### Sequence analysis, genetic variability and evolutionary dynamics of viral populations

HIV-1 viral replication is inevitably linked with viral diversity and evolution^27^. Therefore, we retrospectively explored genetic evolution in HIV-1 DNA samples from the study subjects during the last 15 years. A total of 86 *env* sequences from seven different time points over 14 years were obtained from EEC-3, and 94 *env* sequences from nine different time points over 19 years were recovered from EEC-56. Only two *env* sequences from one out of five time points were obtained from EEC-9 over 15 years. All *env* sequences corresponded to R5-tropic viruses according to PSSM genotypic test. Of all the 182 recovered sequences, only two from one sample in EEC-3 were hypermutated, compared to greater numbers found in other HIV-1-infected subjects or individuals on ART^28,29^ which support the very low viral replication level in these EEC.

We inferred the HIV-1 infection time of the individuals based on the correlation between Spanish *env* sequence divergence and sampling time^17^, generating estimations from 1983 to 1986 (Table 1). These estimations suggest that primary infections occurred five to six years before diagnosis and thus these subjects may have been infected for more than 30 years.

In addition, we intended to obtain near full-length genome sequences from proviral DNA. After 337 amplification attempts with 12.4 × 10^6^ CD4^+^ cells and 6.4 × 10^6^ PBMCs, only 11 were positive, 9 from patient EEC-3 and 1 for each of EEC-9 and EEC-56. Of these 11 positives, only one complete genome was recovered in EEC-3 (GeneBank MN055643). This sequence showed a G > A deleterious mutation in the major splice donor site in position 290 that suppose a very limited replicative capacity of the virus^30,31^. The remaining sequences (92%) showed important deletions throughout the genome mostly in *pol* to *env* genes. These data supported the notion that most provirus in these individuals were defective. Altogether, these results show that this group of EEC has very limited levels of viral persistence in blood, with an extremely low HIV-1 DNA reservoir in peripheral CD4^+^ T cells with no capacity to generate viral mRNA, viral particles or replication competent virions.

**Figure 2:**
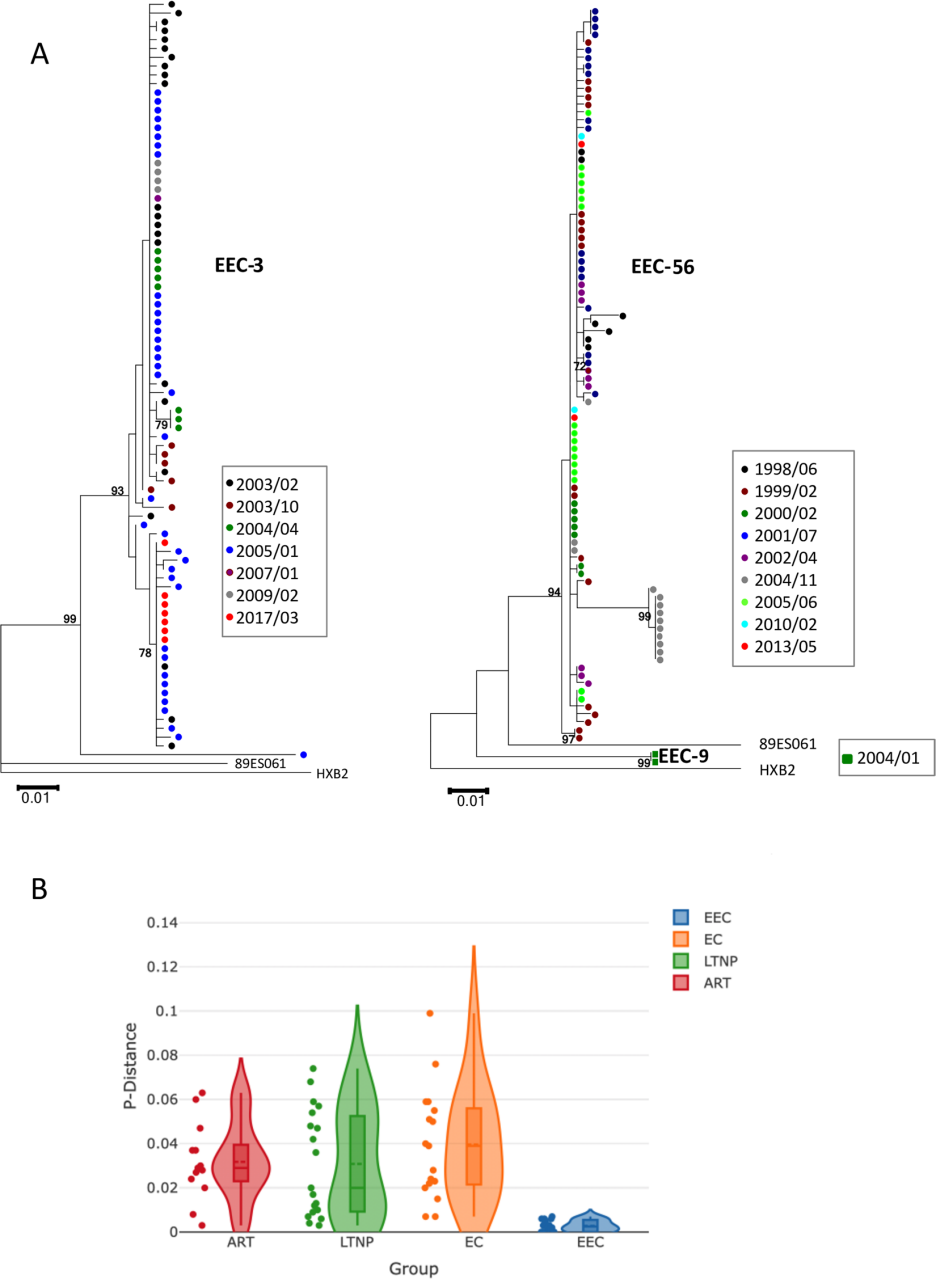
Genetic variability and evolutionary dynamics of viral populations (**A**) Phylogenetic trees with *env* gene sequences of the individuals during follow-up. The evolutionary history was inferred in trees by using the Maximum Likelihood method based on the General Time Reversible model. The percentage of trees in which the associated taxa clustered together with values over 70% is shown next to the branches. Initial tree for the heuristic search was obtained by applying the Neighbor-Joining method to a matrix of pairwise distances estimated using the Maximum Composite Likelihood approach. A discrete Gamma distribution was used to model evolutionary rate differences among sites (4 categories). The tree is drawn to scale, with branch lengths measured in the number of substitutions per site. Evolutionary analyses were conducted in MEGA6. Different colors were used to indicate the sampling time. (**B**) Genetic variability analysis of samples from different groups of HIV-1 individuals with a controlled infection (see Materials and Methods).

The phylogenetic trees showed very short branches with the major presence of viral sequences with no distance to the most common recent ancestor (Fig. 2A). This pattern was already evident in the first sample and it was maintained in different samples over the years. There were isolated small clusters of sequences showing longer branches in EEC-3 and EEC-56, which were not hypermutated. However, these viral populations were evolutionary dead ends and did not appear in further samples.

Moreover, the genetic variability of the viral quasispecies showed a very restricted genetic diversity, estimated to be around 0.010 ± 0.003 substitutions per nucleotide (s/n) throughout the entire follow-up (Fig. 2B). The degree of e*nv*-based heterogeneity was up to eight times lower in EEC than in other HIV-1-infected groups with controlled viral replication, including subjects on ART, viremic LTNP or transient EC one year before the loss of control (FDR < 0.05) (Fig. 2B). Altogether, the practically null viral genetic evolution and extremely low complexity of the viral populations support the absence of viral replication in our EEC group over 30 years.

### Evolution of immune responses

#### HIV-1-specific T-cell responses and poly-functionality

**Figure 3:**
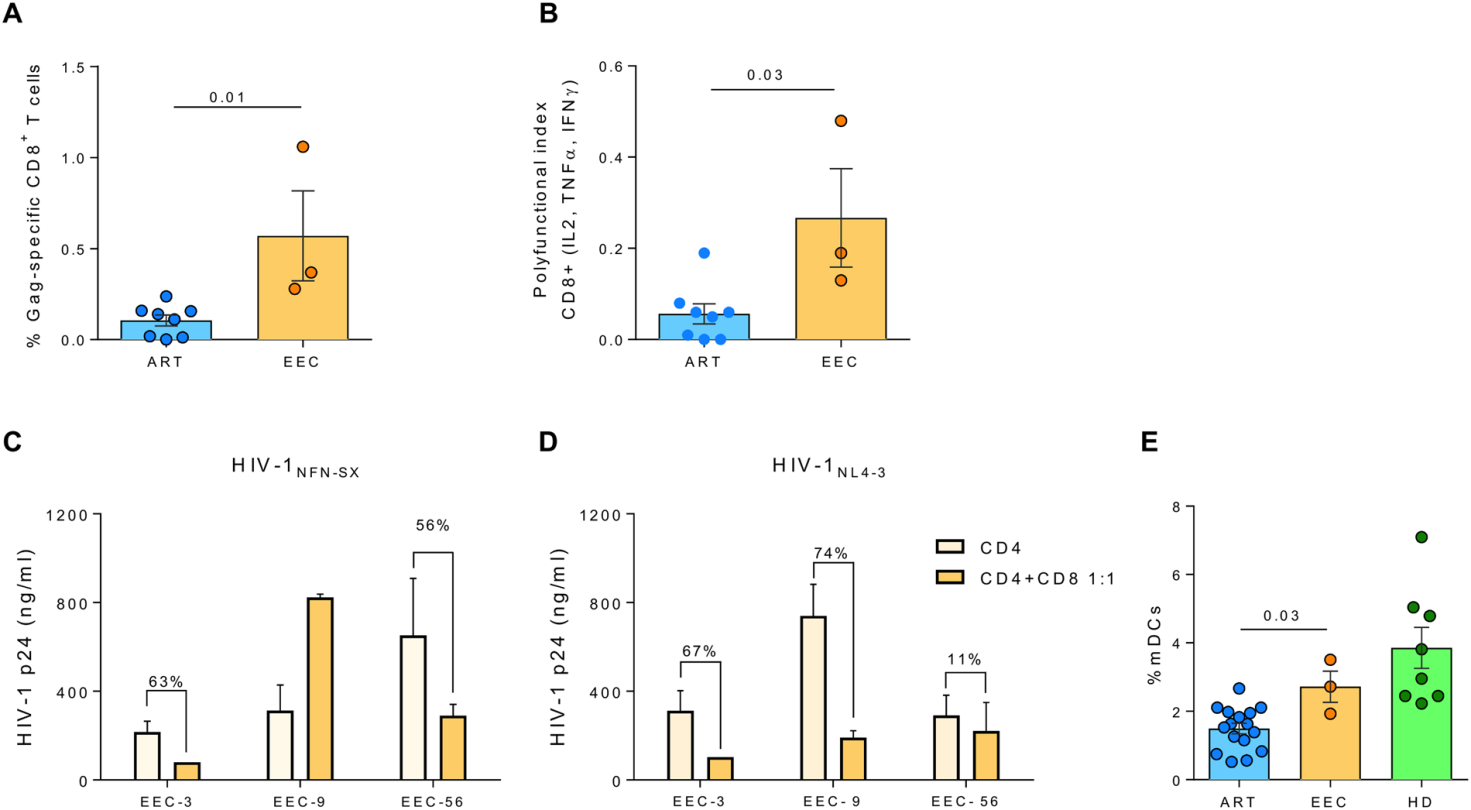
Cellular Immune responses. (**A**) Total CD8^+^ T-cell Gag-specific response from EEC and HIV-1-infected individuals on suppressive ART. (**B**) INDEX of polyfuncionality (pINDEX) of Gag-specific total CD8+ T-cells from EEC and HIV-1-infected individuals on suppressive ART based on the proportions of cells producing intracellular combinations of IFN-γ, TNF-α, IL-2. (**C**,**D**) Viral inhibition assay. Assay of the *ex vivo* ability of CD8^+^ T cells to inhibit superinfected autologous CD4^+^ T cells of the three individuals. The figure shows day 7 of an infection with a laboratory viral strain (**C**) HIV-1_NFN-NX_ (CRR5-tropic) and (**D**) HIV-1_NL4–3_ (CXCR4-tropic). Percentage of inhibition of CD4^+^ vs CD4^+^:CD8^+^ T cells is indicated in each individual. (**E**) Total myeloid dendritic cell quantification, comparing EEC with HIV-1-infected individuals on ART and non-HIV-1-infected healthy donors (HD). (**A**,**B** and **E**) Differences between groups were determined by Mann-Whitney U test.

The magnitude of Gag-specific T-cell response was calculated as the percentage of Gag-specific CD8+ T-cells producing at least one intracellular cytokine (TNFα, IL-2 and/or IFNγ). EEC presented higher levels of Gag-specific total CD8^+^ T-cells when compared with HIV-1-infected individuals on suppressive ART (Fig. 3A). Such differences were also detected in Central Memory (CM), Effector Memory (EM) and Terminally Differentiated (TD) CD8^+^ T-cell levels (Supplementary Information. Figure 2A,C). Simultaneous release of multiple cytokines was determined using the poly-functionality index (pINDEX). The pINDEX in Gag-specific total CD8^+^ T-cells in EEC was higher in three-, four- and five-functions (p = 0.017, p = 0.022, and p = 0.008 respectively) when compared with subjects on suppressive ART (Fig. 3B and Supplementary Information. Figure 2D,E). These values were clearly higher than those of previously published with transient EC one year before the loss of control^10^.

#### CD4^+^ susceptibility and CD8^+^ viral inhibition

To analyze the susceptibility of the target cells to HIV-1 infection, we pulsed autologous CD4^+^ T cells from each individual with laboratory-adapted R5- and X4-tropic viral strains for 7 days (Fig. 3C,D). This assay shows that CD4^+^ T cells from the individuals were susceptible to HIV-1 infection. When we added autologous CD8^+^ T cells to these co-cultures, we observed a reduction of viral replication from 11 to 74% with the X4-tropic virus and up to 63% with the R5 tropic virus (Fig. 3C,D). Thus, host CD4^+^ T cells are not intrinsically refractory to HIV-1 infection with R5 or X4-tropic viruses, and that host CD8^+^ T cells are effective in suppressing viral replication *ex vivo*.

#### Myeloid cell quantification

We also compared innate immune cellular subsets from EEC with HIV-1-infected individuals on ART and with non-HIV-1-infected healthy donors (HD) who were matched with EEC by sex and age. EEC had increased levels of total peripheral mDCs compared with subjects on ART (Fig. 3E), while no statistical differences were observed comparing EEC with HD. Similar results were obtained comparing the levels of myeloid subsets CD1c^+^ and CD141^+^ (Suppl. Figure 2F,G). No differences were observed in pDCs (data not shown). Thus, EEC showed more preserved levels of CD1c^+^ and CD141^+^cells than subjects on ART, and similar levels than sex/age-matched HD and other EC^32^.

#### Inflammation biomarkers

**Figure 4:**
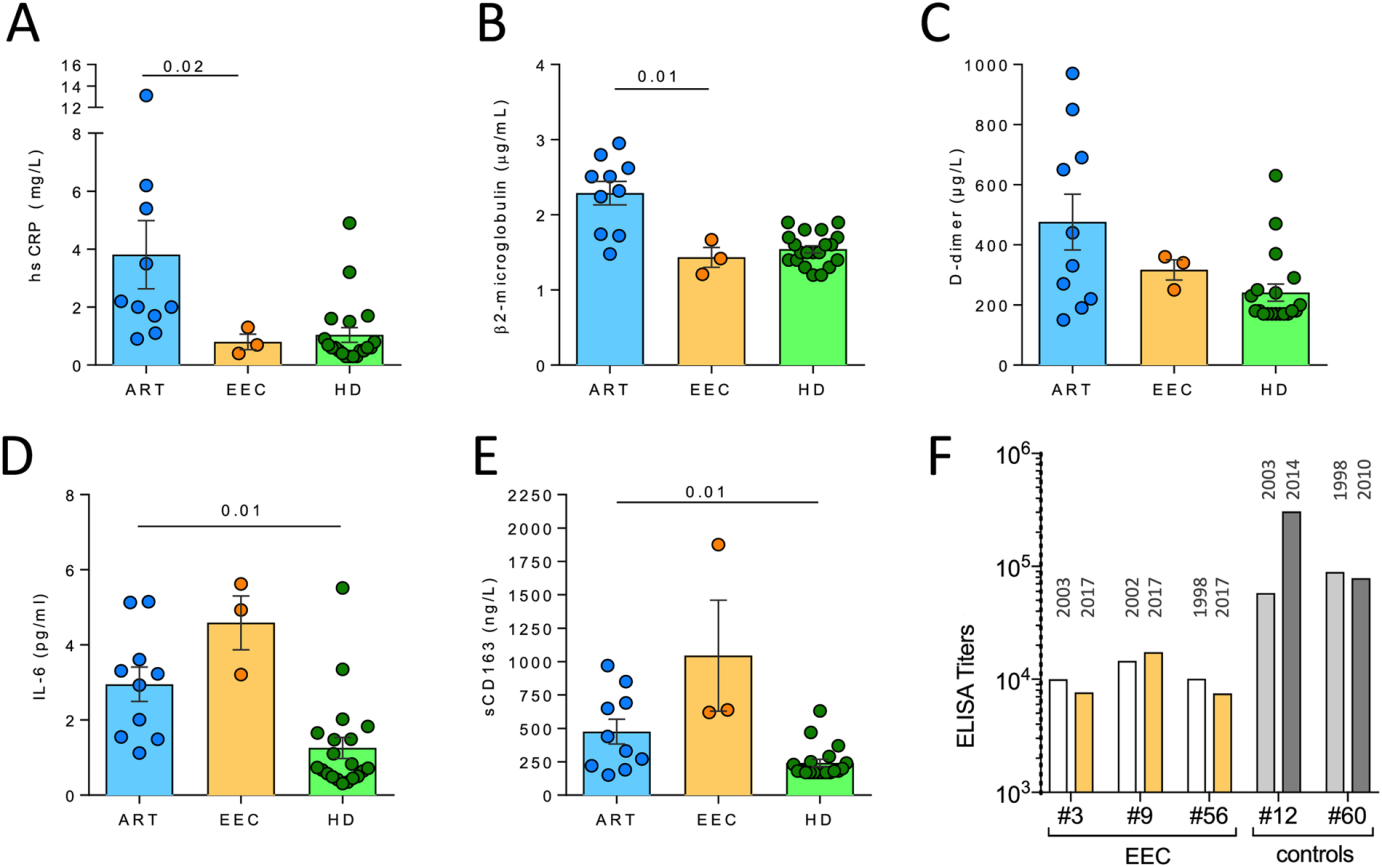
Inflammation biomarkers and antibody titers. (**A**–**E**) Different inflammatory markers were analyzed including (**A**) hsCPR, (**B**) β2-microglobulin, (**C**) D-dimer, (**D**) IL-6 and (**E**) sCD163. EEC individuals were compared with HIV-1-infected individuals on ART and non-HIV-1-infected healthy donors (HD). (**F**) Plasma antibody titers, expressed as the inverse of the final serum dilution with a positive signal, in two samples 14 to 19 years apart.

EEC had lower levels of hsCRP, β2-microglobulin and D-dimer levels than subjects on ART, but comparable to HD (Fig. 4A–E). IL-6 and sCD163 were similar between EEC and ART groups, despite the levels of IL-6 and sCD163 were higher than in HD, probably due to previous history of HCV and residual expression of HIV antigens.

#### HIV-1-specific antibody levels

All EEC included in this study had detectable antibodies against all viral proteins in the LIA-scan analysis and western blot analysis. However, HIV-1-specific antibody levels, determined by ELISA over 14 to 19 years of follow-up, were consistently lower (p = 0.0040) than in viremic LTNP controls (Fig. 4F). Participants displayed a low, albeit persistent, neutralizing response against a mini panel of isolates (data not shown).

## Discussion

Herein we characterize three exceptional elite controllers who spontaneously controlled HIV-1 replication and its associated clinical pathogenesis for more than 25 years. According to our observations, the almost total absence of viral diversity and evolution analyzed during at least 15 years, probably due to a replication-impaired viral reservoir, adds to previously described protective host genetic factors and effective immune responses.

All three EEC individuals included in this study showed a consistently low viral DNA reservoir in peripheral CD4^+^ T cells that was 50 times lower than in individuals on ART using the most sensitive techniques^26^. Total HIV DNA, cell-associated HIV RNA, residual plasma viremia, and IUPM were also lower than other values independently reported for EC^33,34^. Moreover, HIV-1 *env* sequences had nearly null viral genetic evolution, extremely low complex populations and ancestral dating supporting the absence of viral replication over three decades. This replication impairment is further supported by the variation in only two nucleotides between the only complete genomic sequence of EEC-3 and another one obtained 12 years earlier^15^. The lack of sequence heterogeneity despite a long-term untreated infection might reflect a combination of a slow-evolving virus and the clonal expansion of infected immune cells^35^ as suggested by the presence of identical viral clusters in the phylogenetic trees. Finally, the low levels of proviral DNA seemed to be largely defective (>92%), since they had no capacity to generate viral mRNA, viral particles or replication competent virions, implying limited viral persistence in these EECs. The lack of viral replication, as shown by different markers (Fig. 1D–G) and low hypermutation, despite detectable HIV-1 DNA has been mainly associated with the presence of proviral defective genomes and the stability of the viral reservoir^15,16,36^.

Low population size and viral diversity are associated with low replication and viral fitness^37^. These viral characteristics favour important fitness losses, because of the irreversible accumulation of deleterious mutations, known as the Muller´s ratchet effect as observed “*in vitro*”^38^. The combination of these factors is able to prevent viral fitness recovery, and even if some remaining virus is present in the body, it will not generate replication competent viruses because viral populations do not have enough variability to recover fitness^39^. The low diversity and population size could have contributed to the maintenance of the control of viral replication in the participants during the follow-up. All study subjects showed the lowest viral diversity values among comparative groups of subjects on ART or LTNP with viral load control. In addition, in a previous study we showed that low diversity and lack of viral evolution was highly correlated with permanent viral control in persistent EC^10^. On the contrary, transient EC who lost control during the follow-up showed higher levels of genetic diversity^10^ (Fig. 2B). Thus, extremely low viral diverstiy could be a convenient prognostic marker for the identification of these EEC.

The functional characterization of viruses from EEC has been limited by the impossibility of complete virus recovery. In a previous study, EEC-56 showed the major presence of 228 nucleotide deletions in the 5′ LTR–gag region; in EEC-3 a 247 nucleotide deletion was positioned in *pol* gene up to the vif orf^15^. These deleted genomes became dominant during follow-up^15^. In other studies, we could clone *env* sequences from EEC-3 in recombinant viruses that displayed a very limited and retarded replication capacity^40,41^, similarly to what has been observed in *env* recombinant viruses from some EC^42^. Pseudoviruses with *env* clones from EEC-3 exhibited a very low CD4 binding, transfer and fusion capacity, and a very inefficient cell signaling resulting in a very restricted replicative capacity^41^.

According to the data reported in this work, viral antigens and/or truncated viral proteins could be generated in these individuals from defective genomes or from new alternative spliced HIV-RNA variants with translationally competent ORFs as reported in cART subjects^43^. The apparent limited amount of peripheral viral antigen might be associated with the observed low, albeit detectable, levels of HIV-1-specific antibodies, including neutralizing antibodies (data not shown). However, such levels were still superior to those of the two long-term described cases of HIV-1 cure after allogeneic stem cell transplant with a CCRΔ32 donor^4,5^. Likewise, both frequency and poly-functionality of Gag-specific CD8^+^ T-cell responses were higher in EEC when compared with subjects on ART (Fig. 3A) and similar or above the median of a group of persistent EC (with more than 18 years of controlled infection) and higher than those of transient EC^10^. The CTL responses supported the capacity of the immune system to mount effective adaptive immune responses as confirmed by the *ex vivo* viral inhibition assay. Inhitibion from autologous CD8^+^ T cells was similar to previous described EC and higher than comparisions with non-HIV infected donors^44^. Innate immune responses seem to be relatively normalized as for the quantification of inflammation biomarkers and frequency of myeloid cells when compared to HD individuals. Only IL-6 and sCD163 were slightly superior compared to HD to subjects on ART. Values shown in Fig. 4A–E were in the range of those found in indepentdent cohorts of EC^32,45^. This could be due to the previous long-term story of HCV infection, and in fact, sCD163 has been proposed as a marker of liver fibrosis^46^. This is a substantial difference with previously published EC profiles where despite the viral control, inflamation levels were maintained over time^10,47^. Whether this could be a distinctive marker easy to assay for EEC needs to be examined in extended populations, but previous works with persistent EC suggested this idea^10,48^. The interplay of T cells with the preserved number and functions of innate cells might have been a critical factor for the maintenance of the high HIV-1-specific T-cell responses in these subjects^49^. Although there is sligth decline in CD4 + T-cell levels in EEC-9, this decrease can be considered physiological with aging, during the 20 years of follow up and with the fact that the studied subjects are now over 60 years old, and moreover this diminution is comparable with European uninfected individuals^50^.

We can only speculate how these HIV-1-infected subjects have resulted in this EEC phenotype of potential functional viral suppression. Thus, primary infection might have occurred with a low fitness viral founder strain, or alternatively, initial innate immune responses might have shaped the selection of an unfit virus. Host genetic factors, including those related with the HLA function, and cellular-adaptive immune responses might have further contributed to this clinically unusual non-progressive profile. Although these are naturally occurring cases, and not the result of a clinical intervention, they provide knowledge on how to achieve a functional cure for HIV-1 infection. Based on all these evidencies we believe that these individuals do not require any ART. Perhaps other individuals, most probably within the persistent EC, LTNP-EC, and EEC groups with similar host (at least three to four alleles), inmune responses (with a polyfunctionality index > 0.50) and viral factors (with stable viral diversity (below 0.010 ± 0.003 (s/n) throughout the entire follow-up) could also have spontaneously achieved a HIV-1 functional cure^51^.

Four previous cases of durable control over HIV replication without ART have been reported^12^. While those cases also had extraordinarily low HIV burdens, they differed from ours in their shorter follow up (median of 9 years since HIV diagnosis) and their weak reactive Western blots. A more recent study in HIV-1 Controllers (HIC) followed up for a median of 21 years has shown a similar small HIV blood reservoir in the presence of weak T-cell activation levels^11^. Our study adds three new additional characteristics to this EEC phenotype: (i) even more time since HIV diagnosis without ART, (ii) low viral diversity and lack of viral evolution over the years, and (iii) inflammatory markers in peripheral blood similar to those in healthy donors.

The incidental accumulation of protective host factors, unfit and/or defective viruses that precluded viral evolution and diversification, effective innate and adaptive immune responses, might have take place simultaneously to achieve a HIV-1 functional spontaneous cure. This model of control suggests that new curative strategies would be successful combining approaches that achieve a reduction of the HIV-1 latent reservoirs, maintain extremely low viral diversity, an impaired viral fitness, and enhanced HIV-1 specific immune responses.

## Supplementary information

Supplementary information.
