# Supplementary material for: Permanent control of HIV-1 pathogenesis in exceptional elite controllers: a model of spontaneous cure
Source: Sci Rep. 2020 Feb 5;10:1902. doi: 10.1038/s41598-020-58696-y (PMC7002478; doi:10.1038/s41598-020-58696-y)

Supplementary Information.

Permanent control of HIV-1 pathogenesis in exceptional elite controllers: a model of spontaneous cure

Concepcion Casado[1], Cristina Gálvez [2,3], Maria Pernas[1], Laura Tarancón-Diez[4], Carmen Rodríguez[5], Víctor Sánchez-Merino[6], Mar Vera[5], Isabel Olivares[1], Rebeca De Pablo-Bernal[4], Alberto Merino-Mansilla[6], Jorge Del Romero[5], Ramon Lorenzo-Redondo[7], Ezequiel Ruiz-Mateos[4§], María Salgado [2,§], Javier Martinez-Picado [2,8,9,§,*], Cecilio Lopez-Galindez [1,§,*]

Supplementary Figure 1. **Schematic diagram of the flow cytometry gating strategy.**

For (A) Gag-specific CD4$^+$ and CD8$^+$ T-cell. Respresentative plots show the functional cytokine response to Gag peptides and (B) For dendritic cells subsets.

Supplementary Figure 2. Cellular immune responses

(A-C) CD8$^+$ T-cell Gag-specific response. (A) Central Memory, (B) Effector Memory and (C) Terminally Differentiated Gag-specific CD8$^+$ T-cell levels from EEC and HIV-1-infected individuals on suppressive ART. (D-E) INDEX of Polyfuncionality (pINDEX) of Gag-specific total CD8+ T-cells from EEC and HIV-1-infected individuals on ART based on the proportions of cells producing intracelular combinations of IFN-γ, TNF-α, IL-2 (D) plus CD107a (4 functions), and (E) plus perforin (5 functions). (F-G) Mieloid cell quantification, (F) CD1c$^+$ and (G) CD141$^+$ levels comparing EEC individuals with HIV-1-infected

individuals on ART and non-HIV-1-infected donnors (HD). (A-G) Differences between groups were determined by Mann-Whitney U test.

Supplementary Figure 1

A

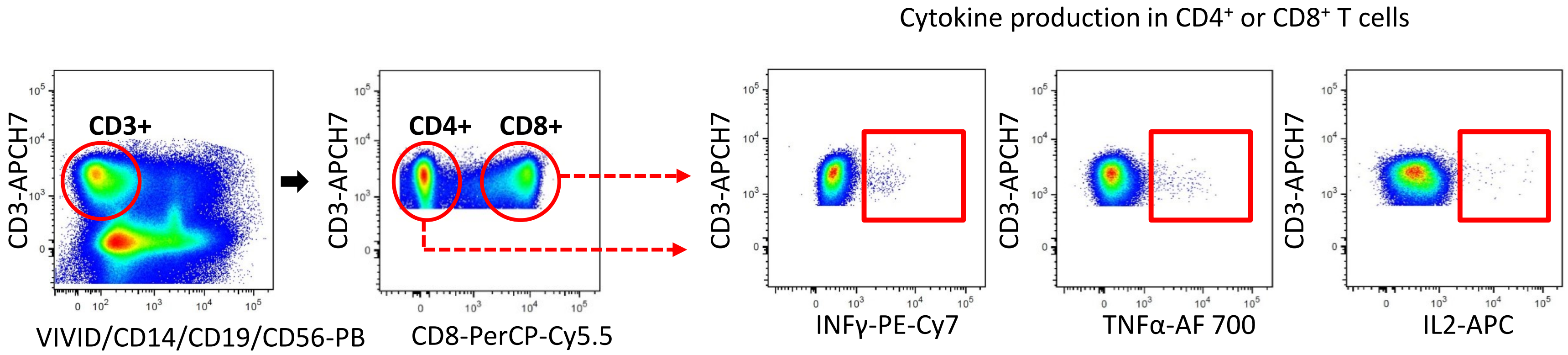

B

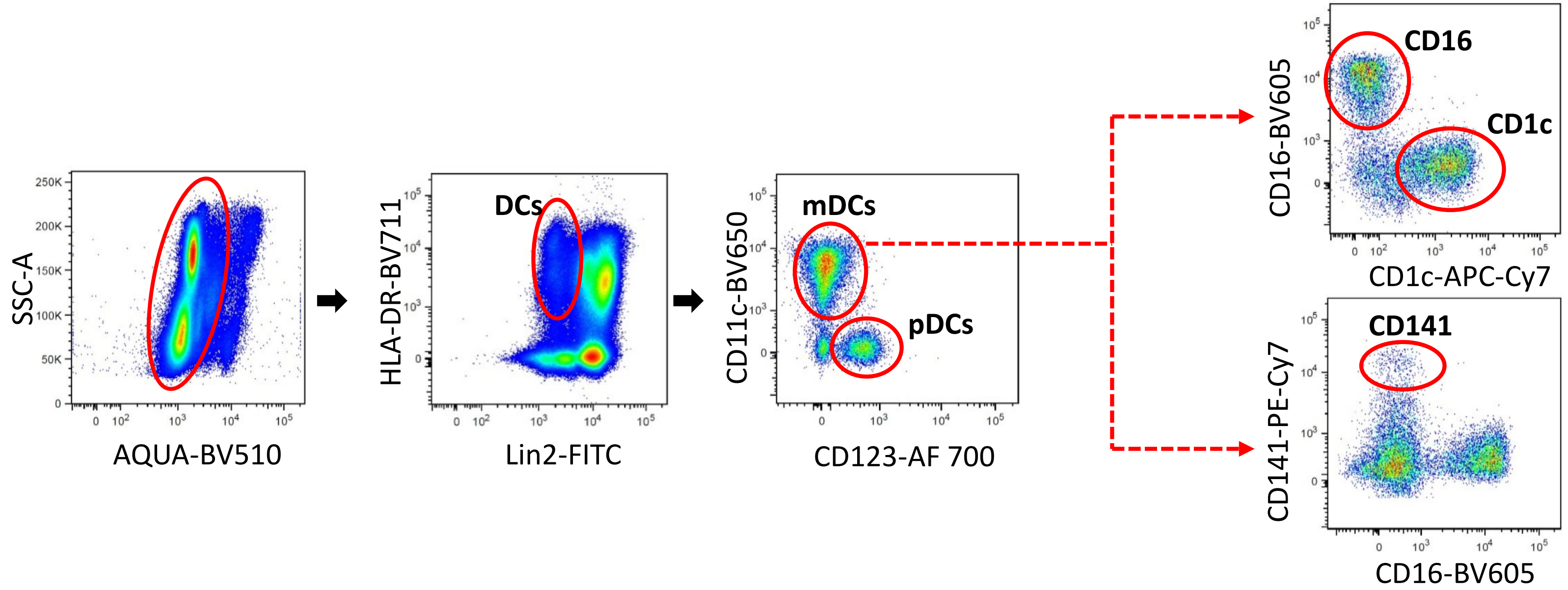

Supplementary Figure 2

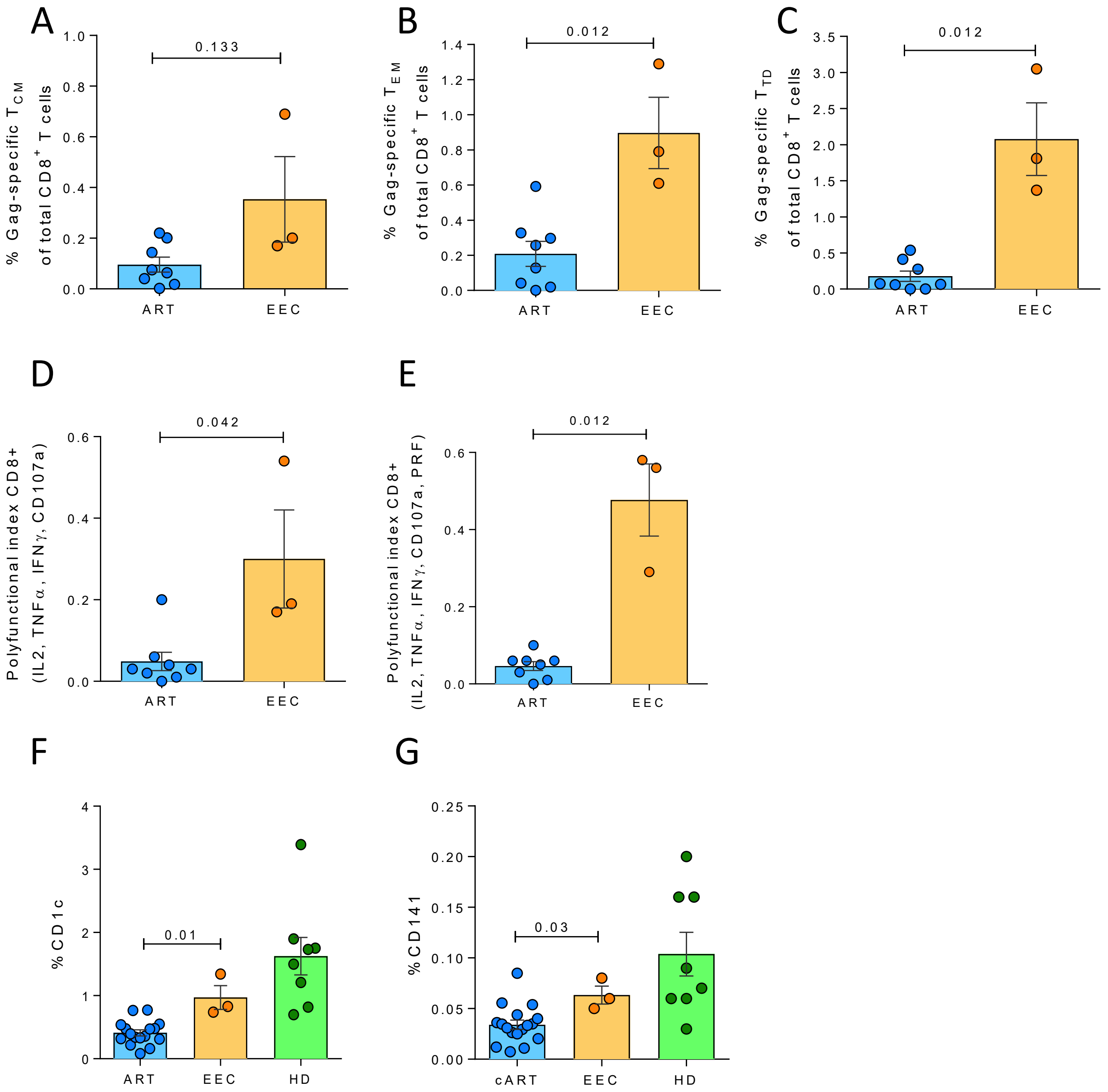

Supplement: Supplementary file 1 — Supplementary information. [file 41598_2020_58696_MOESM1_ESM.pdf]
